# Social Network Characteristics and Salivary Cortisol in Healthy Older People

**DOI:** 10.1100/2012/929067

**Published:** 2012-03-12

**Authors:** Julian C. L. Lai, Alice M. L. Chong, Oswald T. Siu, Phil Evans, Cecilia L. W. Chan, Rainbow T. H. Ho

**Affiliations:** ^1^Department of Applied Social Studies, City University of Hong Kong, Hong Kong; ^2^SCOPE, City University of Hong Kong, Hong Kong; ^3^School of Social Sciences, Humanities and Languages, University of Westminster, London W1B 2UW, UK; ^4^Center on Behavioral Health, University of Hong Kong, Hong Kong

## Abstract

Psychobiological research on aging in humans has been confounded by individual differences that have not been adequately characterized in the literature. This paper is an attempt to shed light on this issue by examining the impact of social network characteristics predictive of successful aging on salivary cortisol among 78 older Chinese people in Hong Kong. Eight salivary cortisol samples were collected each day for two consecutive days from immediately after awakening to 12 hours later. Two components of the cortisol diurnal cycle, response to awakening and diurnal decline, were examined in relation to social network characteristics including size, emotional support, and cultivation. ANOVAs with repeated measured were run to examine influences of the three social network characteristics on the cortisol awakening response and diurnal decline, with the effects of gender, age, socioeconomic status, and waking time controlled. Results indicated that those who spent more time and effort in developing and strengthening their social ties (i.e., those high in “cultivation”) exhibited a significantly greater rise in cortisol in the morning and a significantly steeper decline over the day, thus attesting to more effective activation and deactivation of the HPA axis. Network cultivation reflected a positive motivation to nurture social relationships more than the other two network characteristics. Its effect on cortisol might stem from the positivity underlying the motivation.

## 1. Social Network Characteristics and Salivary Cortisol in Healthy Older People

The last two decades have witnessed a proliferation of research on the impact of psychosocial factors on cortisol secretion in aging populations, which has been motivated by the assumption that the capacity to adapt to stressful forces in life declines with age and this is marked by a progressive dysregulation of the hypothalamic-pituitary-adrenal (HPA) axis [1, 2]. Being the endproduct of the HPA axis, cortisol has been extensively studied in relation to the aging process [3–5]. There is evidence showing that basal cortisol levels (e.g., [6, 7]) and reactivity to psychosocial and pharmacological challenges increase with age (e.g., [8, 9]). Despite the significance of these findings, their interpretability is moderated by mixed findings (e.g., [10, 11]) and a gender effect reported in a number of studies (e.g., [8]). This may partially reflect the remarkably heterogeneous responses of the aged population to stressful challenges, which have not yet been adequately characterized. It is possible that some old people may age more successfully than others [12] and the two sexes cope with daily stressors and distress differentially [13].

One of the most challenging tasks in studying the impact of aging on cortisol is the development of reliable markers of cortisol secretory activity because the HPA axis is highly adaptive and is characterized by marked inter- and intra-individual variability. Cortisol exhibits a robust diurnal rhythm, peaking within 60 minutes after waking and declines thereafter until reaching the nadir around midnight [14]. Recent evidence suggests that the cortisol awakening response (CAR), which is marked by an increase from 50% to 150% within the first 30 minutes after waking up in the morning [15], is a reliable marker of HPA axis functioning [16].

This distinct component of the cortisol diurnal rhythm has a salient genetic component [17]. An absence or accentuation of the CAR is associated with various psychopathological conditions or adverse outcomes (e.g., [18–21]). On the other hand, attenuation in postawakening cortisol levels has been observed in people who are high in optimism [22] or experience more positive affect [23].

Another reliable marker of the integrity of the HPA axis is the diurnal variation or decline which has been operationalized as a descent of cortisol levels measured at different times over the day [24, 25]. Variations in this diurnal profile have been shown to be related to a number of clinical conditions [25]. In particular, a flatter profile has been observed in people with depression [26], breast cancer [27], PTSD [28], and under severe parenting stress [29].

Despite the demonstrated sensitivity of the cortisol diurnal rhythm to psychosocial factors and clinical conditions, it has not attracted due attention in geriatric research until recently. Recent studies have shown that older participants exhibit a diurnal pattern marked by a lower CAR and a flatter slope of diurnal decline [30, 31]. This flatter diurnal profile in cortisol has also been found to have adverse health implications [32, 33] There is also evidence showing that postawakening cortisol is influenced by a number of psychosocial factors in the elderly. For example, higher socioeconomic status [34], higher wellbeing [35], and higher coping humor [12] have been found to be associated with lower levels of postawakening cortisol. 

In sum, the evidence reviewed earlier show that aging is associated with an attenuated CAR and a flattened diurnal profile. In addition, positive psychological factors may predict lower cortisol levels in the awakening period. Keeping these issues and findings in mind, we designed the present study to examine the impact of psychosocial factors predictive of successful aging [36] on components of the cortisol diurnal cycle in older Chinese people. 

We focused on variables pertaining to social networks on the basis the following considerations. Firstly, according to the socioemotional selectivity theory [37, 38], social networks, especially those rich in capital, through the support they provide, contribute positively to the well-being of older adults. Although not all types of support derived from social networks are uniformly beneficial to all older recipients [39], the weight of findings from research in the last few decades tends to support the aforementioned conjecture [40]. 

Secondly, there is a huge volume of evidence showing that social integration is relevant to successful aging (e.g., [41, 42]). Numerous studies have shown that a higher degree of social integration, indexed by various measures of social networks, is consistently associated with better mental and physical health (e.g., [40]), lower likelihood of dementia [43], and lower mortality [44] in the elderly. Among social network characteristics that have been studied, size or the number of social ties and emotional support appear to be most important in terms of the positive effects that they have on health and other measures of functional status in older populations (e.g., [39, 45], although their impacts have not been demonstrated in a handful of longitudinal studies (e.g., [46, 47]).

Thirdly, cortisol has been shown to be sensitive to variation in social support in a number of studies, which lends additional support to the health implications of social network characteristics. For instance, lower social support in older people has been found to predict a lower AUC in the diurnal profile of cortisol [30]. In younger populations, there is evidence showing that individuals having higher social support showed lower basal cortisol levels [48], attenuated cortisol reactivity [49], and faster recovery in response to laboratory stressors [50]. In another recent study, Pressman et al. [51] found in college students that a small social network was associated with suppressed immune responses to influenza vaccination. Although cortisol was not examined in this study and thus it is not certain whether it mediated immune effects, there is substantial evidence implicating cortisol in the regulation of immune responses [52], taken together, these findings suggest that support derived from social networks affects cortisol levels and this may have significant health implications.

Fourthly, the importance of social networks to successful aging has also been demonstrated recently in Chinese elderly in Hong Kong [53]. These investigators studied three characteristics of ego networks, namely, size, support (both emotional and instrumental) and cultivation in a community sample of 2970 Chinese older adults. The last characteristic refers to time and efforts committed to the maintenance and strengthening of existing social ties, which has not been systematically studied previously. Although the propensity to connect with others (PCO) has been shown to be associated with well-being positively [54], this concept focuses on making connections with strangers and is conceptually different from the tendency to strengthen existing relationships with others. We believe that cultivation of social ties should be as crucial as conventional network characteristics in determining health and well-being in the elderly because the socioemotional selectivity theory predicts that older people will receive the support that they need from a small number of ties in which they invest most of their time and efforts to maintain and cultivate [37]. In line with this, Chong et al. [53] found that efforts exerted in cultivating and strengthening social ties (*cultivation*) emerged as a significant predictor of successful aging in Chinese older adults.

To sum up, findings and theories reviewed earlier suggest that social network characteristics are relevant to successful aging and may affect cortisol levels. Moreover, it has also been clearly demonstrated that the CAR attenuates and diurnal profile of cortisol becomes flatter with age. On the basis of these findings, we hypothesized that social network characteristics, especially the motivation to strengthen existing social ties (*cultivation*) would be significantly associated with the diurnal rhythm of salivary cortisol in older Chinese people. Specifically, those with a stronger motivation in *cultivation* would exhibit a less attenuated CAR and a significant diurnal decline in cortisol in comparison with their peers with a lower motivation in *cultivation*.

## 2. Method

### 2.1. Participants and Procedures

Eighty-four Chinese community dwelling older individuals (45 men) took part voluntarily in the study. They were retirees recruited from a number of senior or community centers with which one of the authors had contact. Each of them was given HKD100 (in the form of a shopping coupon) in return for participation. Their ages ranged from 59 yrs to 86 yrs (mean age = 73.0 yrs). Participants were free of heart diseases, cancer, and psychiatric illnesses, and were not on medication such as estrogen, synthetic glucocorticoids, antisteroid drugs, and antiseizure drugs that would potentially affect cortisol concentrations. In addition, all participants reported that they did not smoke. Ethical approval was obtained from the relevant approving body of the City University of Hong Kong for conducting the study.

All participants were briefed by the experimenter in the first home visit during which they were given a study pack containing fully standardized written instructions, questionnaires, saliva sampling tubes (Salivettes), a comprehensive description of the procedure of the study including instructions for using the Salivettes and an electronic timer used to control the timing of saliva collection. The electronic timer is a simple device that can be used to alert participants with an auditory alarm immediately after the countdown of a predetermined period of time. Participants were asked to provide saliva samples according to a specific protocol for two consecutive days. They were instructed to collect one saliva sample using the Salivette (Sarstedt AG & Co., Nümbrecht, Germany) on each day at each of eight collection times: immediately after awakening, 15, 30, 45 minutes, 3, 6, 9, 12 hours postawakening. To ensure that the last four samples would be collected in accordance with the protocol, participants were asked to adjust their activities so that these four samples must be collected at home on time. Moreover, participants were asked not to eat and drink anything other than water and refrain from brushing of teeth during the awakening period. For the remaining samples, participants were asked to refrain from eating and drinking anything but water at least 30 minutes before taking each sample. The correct use of the timer was also explained to ensure that saliva samples would be collected on time.

Saliva samples were stored in the participants' home freezers until the second home visit during which the saliva samples were collected and participants were asked to complete a questionnaire collecting demographic and psychosocial information. As the English literacy of the participants was not expected to be adequate, the questionnaire was written in Chinese. The saliva samples were stored at −20°C in the laboratory until they were thawed for biochemical analysis. Data collection was completed within 3 months to minimize seasonal impact on cortisol concentrations.

### 2.2. Cortisol Assay

The biochemical assay was conducted at the laboratory of the Center on Behavioral Health of the University of Hong Kong, using a procedure adopted in prior studies [12, 22, 55]. Saliva samples were thawed and centrifuged at 3000 rpm for 15 minutes at room temperature. Clear supernatant was used for analysis. Cortisol levels were determined by using an enzyme-linked immunosorbent assay kit (ELISA) developed for use in Saliva (Salimetrics, LLC, State College, PA, USA). The assay sensitivity for the kit was 0.2 nmol/L. Intra-assay and inter-assay coefficients of variation were 3% and 10%, respectively.

### 2.3. Measures

#### 2.3.1. Social Network Characteristics

Social networks refer to the totality of social relationships a person develops over time via interactions with people who constitute the main source of companionship, sharing, and support, through which the person satisfy his or her psychological and social needs [56]. Social network characteristics were assessed by the Social Network Scale (SNS) developed by Chong et al. [53] to assess three characteristics of social networks, namely, size, support, and cultivation. *Size* was measured by exploring the size of different types of networks with 4 items in relation to family, relatives, friends, and neighbors, respectively. Respondents indicated the number of most significant persons in each of the four categories of relationship using a 5-point scale (1 = 0, 2 = 1 to 2, 3 = 3 to 4, 4 = 5 to 6, and 5 = 6 or more). *Support* was tapped by a 13-item subscale measuring the use of the aforementioned 4 types of networks for emotional support (4 items), financial support (5 items), and companionship (4 items). Only emotional support was used in the present study because as reviewed earlier, this type of support has been shown to predict wellbeing in the elderly more reliably than instrumental support. Respondents used a 5-point scale to indicate how frequently they sought support from persons pertaining to each of the 4 categories of relationship (1 = never, 2 = rarely, 3 = at times, 4 = often, 5 = always). *Cultivation* refers to the time and efforts invested by respondents to cultivate each of the 4 categories of relationship. It was measured by 4 items using a 5-point scale ranging from 1 = not at all to 5 = very much.

#### 2.3.2. Socioeconomic Status (SES)

This was assessed with an adaptation of a subjective measure used in a recent study [34]. Subjective measures of SES have a number of advantages in assessing SES in the aging populations over conventional measures tapping absolute levels of SES, which have been summarized in Wright and Steptoe. To complete the measure, participants were asked to indicate on a drawing of a ladder of ten rungs where they stood in society. They were told that the top rung represents people who are the best off and the bottom one those who are the most worst off. The mean of this measure was 5.63 (SD = 2.31) for the present sample.

## 3. Results

As in prior studies, the cortisol data of the current sample were highly skewed. Extreme values were winsorized in order to reduce the impact of outliers: large values were winsorized at two standard deviations and at the lower end, 0.2 nmol/L for values small than 0.2 nmol/L [12, 22]. Square root transformation, which has been one of the most reliable methods to reduce data skewness, was applied before subjecting the data to further analysis. The square-root transformation successfully reduced data skewness by lowering the range of skewness statistics from  .37 to 2.20 before transformation to 0 to  .80 after transformation. All analyses carried out on cortisol concentrations were based on the square-root transformed data.

All participants adhered to the protocol on the basis of the records of sampling times that they returned. The mean waking times of the two days were similar, 5:40 am on the first and 5 : 50 am on the second day, respectively, and were also highly correlated, *r* = .93, *P* < .001. With respect to missing data, 83 participants provided a complete set of social network data whereas 75 participants managed to provide all saliva samples. Data from 4 participants who managed to provide no less than 14 saliva samples over the 2 days were retained. Missing values were then filled in by the means. As a result, data from 78 participants (40 men) who provided complete cortisol and psychological data were included in subsequent analyses. The mean age of this final sample was 73.21 years (SD = 6.90), and 73.6 and 72.8 years for men and women, respectively. The main characteristics of this sample are summarized in Table 1.

### 3.1. Diurnal Rhythm of Salivary Cortisol

The mean cortisol concentrations observed in the current sample are comparable to those reported in previous studies with Chinese participants (Table 2) [12, 22]. The product-moment correlations of cortisol concentration at each of the eight sampling times across the two days, though moderate, were significant, with *rs* ranged from .26 to .58. These correlations imply acceptable intraindividual stability across the 2 days, which is similar to that reported in prior studies [15, 22]. The mean morning cortisol levels at the eight sampling times on the first and second day were summarized in Table 2. 

A two-way ANOVA with repeated measures was run to examine the change in cortisol levels across the two days. The Huynh-Feldt epsilon was used to adjust the degrees of freedom when results of the sphericity tests were significant. Results showed that overall cortisol concentrations did not differ between the two days, *F*(1,77) = 1.80, *P* > .05. There was a significant change in cortisol levels within the day, *F*(3.43,442.03) = 117.13, *P* < .001. However, the diurnal profile of cortisol did not differ between the two days, as indicated by a nonsignificant interaction, *F*(5.74,442.03) = 1.22, *P* > .05.

Several composite measures of cortisol levels including the mean of the 4 postawakening samples, the mean of the afternoon samples, the area under the curve with reference to ground [57], and diurnal decline defined by the difference between the first and the last sample [58], were significantly correlated: *r*s =  .61 (*P* < .001), .46 (*P* < .001),   .46 (*P* < .001), and  .59 (*P* < .001), respectively. In view of the stability of these indices and the similarity of secretion pattern across the two days, the cortisol profiles of the two days were averaged to arrive at a more parsimonious presentation. Subsequent analyses were based on this averaged cortisol profile.

### 3.2. Social Network Characteristics

The three social characteristics were only modestly correlated with *rs* ranged from  .36 to  .43, which are comparable to those reported by other investigators (e.g., [46]). The means of *size*, *emotional support*, and *cultivation* were 11.63 (SD = 3.82), 8.60 (SD = 3.22), and 10.56 (3.00), respectively. The three scales exhibited an acceptable level of internal consistency: *size*, *α* = 62, *emotional support*, and *α* = .71; *cultivation*, *α* = 70.

### 3.3. Social Network Characteristics and the Cortisol Awakening Response (CAR)

The CAR was defined by changes across the 4 samples collected during the postawakening period. The impact of the 3 social network characteristics on CAR was examined by using the General Linear Model approach with repeated measures (PASW 18.0), with the effects of age, gender, SES, and waking time statistically controlled. The Huynh-Feldt epsilon was used to adjust the degrees of freedom when results of the sphericity test were significant. Results showed nonsignificant overall effect on cortisol levels in the awakening period, *F*(2.67,186.63) = 2.50, *P* = .07. However, results of within-subject contrast analyses showed that cortisol levels increased in a linear fashion, *F*(1,70) = 4.77, *P* < .05. Post hoc pairwise comparisons were run to further examine this significant linear trend. Results showed that there was a significant rise in cortisol from immediately to 15 and 30 minutes postawakening: *t*(77) = 2.47, *P* < .02, and *t*(77) = 2.34, *P* < .03, respectively. The main effect of each of the three social network variables was not significant: *size*, *F*(1,70) =  .11, *P* > .50; *emotional support*, *F*(1,70) =  .03, *P* > .50; *cultivation*, *F*(1,70) =  .01, *P* > .50. None of the effects associated with sex, age, SES, and waking time was significant. The only significant effect was the interaction between *cultivation* and sampling times, *F*(2.67,186.63) = 3.72, *P* < .05. Given the significance of this omnibus term with *cultivation *entered as a continuous variable, the interaction was explored further by looking at the cortisol profiles of participants above and below the median on *cultivation* (median = 10.5). ANCOVAS were then run separately for the two groups scoring high versus low on *cultivation* with gender, age, SES, waking time, and the other two social network indices as covariates. Results indicated that a significant change in cortisol levels in the postawakening period was observed only in the group with higher scores in *cultivation*, *F*(3,96) = 4.36, *P* < .01. In particular, cortisol level at 15, 30, or 45 minutes postawakening was significantly higher than that at immediately postawakening: 15 minutes postawakening, *t*(38) = 3.05, *P* < .01; 30 minutes postawakening, *t*(38) = 3.76, *P* < .01; 45 minutes postawakening, *t*(38) = 2.56, *P* < .02. For the group with low scores in *cultivation*, a significant change in cortisol levels was not observed, *F*(2.9,92.72) =  .18, *P* > .90. Results of pair-wise comparisons did not show any significant rises in cortisol from immediately postawakening to 15, 30, or 45 minutes postawakening: *t*(38) =  .81, *P* > .50, *t*(38) = −.10, *P* > .90, and *t*(38) = −1.00, *P* > .30, respectively. Changes in postawakening cortisol levels in the two groups were summarized in Table 3.

### 3.4. Social Network Characteristics and Diurnal Rhythm in Cortisol

Diurnal rhythm in cortisol was examined by analyzing the change in concentrations across the first (immediately after waking) and the last four samples (3, 6, 9, and 12 hours postawakening). Analyses applied to examine the impact of social networks on CAR were adopted. Results showed that the change in cortisol levels over the day was not significant, *F*(2.96,207.47) = 1.17, *P* > .30. The main effect of each of the three social network variables was also not significant: *size*, *F*(1,70) = 1.75, *P* > .10; *emotional support*, *F*(1,70) =  .01, *P* > .80; *cultivation*, *F*(1,70) =  .10, *P* > .70. Sex, age, SES, and waking time also had no significant effect on cortisol. However, a significant interaction between change in cortisol and social network cultivation was observed, *F*(2.96,207.48) = 2.93, *P* < .05. Mean levels of cortisol in the two groups with high versus low scores on *cultivation* were summarized in Table 4. Separate analyses of the two groups scoring high versus low in social network cultivation (i.e., above versus below the median of *cultivation*) were conducted to further examine the interaction. It was found that a significant change in cortisol over the day was observed only in the group with higher scores in *cultivation*, *F*(3.75,120.06) = 3.16, *P* < .02. A significant change in cortisol levels was not observed in the group with lower scores in *cultivation*, *F*(2.74,87.63) =  .31, *P* > .80. Diurnal slopes of the two groups were computed by dividing the difference between cortisol level at 30 minutes and 12 hours postawakening by the time interval between these two samples [59]. The group with higher scores on *cultivation* had steeper slopes (M = −.17, SD =  .07) compared to their peers with lowers scores on *cultivation* (M = −.13, SD = .08): *t*(76) = 2.06, *P* < .05.

## 4. Discussion

Findings of the present study highlight the importance of social network cultivation in affecting the diurnal rhythm of salivary cortisol in a sample of healthy older adults. Consistent with our prediction formulated on the socioemotional selectivity theory [37] and findings reported by Chong et al. [53], cultivation of social networks was found to modulate both the CAR and the diurnal cortisol profile. As reported earlier, participants with higher scores in *cultivation* tended to exhibit cortisol profiles with a greater CAR and a more pronounced diurnal decline in comparison to their peers having lower scores in *cultivation*. This diurnal profile is similar to that exhibited in younger people (e.g., [30]) or relatively younger ones in an older sample [31], implying that cultivation of social ties may serve to “rejuvenate” the diurnal cortisol profile in older people. Given that cultivation of social ties has been shown to promote successful aging (e.g., [53]), our findings, which constitute the first set of data linking cultivation of social ties to cortisol, will further substantiate the role played by cortisol in determining health and function status in later life.

Conventional network characteristics such as size and emotional support did not significantly influence cortisol levels in either the postawakening or diurnal sampling period. The important implications of these findings become apparent if we recall the evidence, presented earlier, which showed that the most important factor determining longevity prospectively in the elderly was the quality of social ties rather than size of networks (e.g., [44]). In a similar vein, Pinquart and Sörensen [41] have also demonstrated the importance of quality of social contacts in determining subjective wellbeing in later life. These findings are in line with the prediction of the socioemotional selectivity theory because the size of networks tends to shrink when people become older and satisfaction of their psychosocial needs may be derived from a limited number of ties most significant and meaningful to them. Although more emotional support has been shown to benefit wellbeing and functional status in the elderly in a number of cross-sectional studies (e.g., [45]), its effect seems to be conditioned by factors which have yet to be understood more completely. For instance, emotional support has not been found to confer protection against decline in physical functioning over a period of 7 years [47]. Moreover, more emotional support has been shown to predict poorer cognitive and functional status longitudinally [46]. In other words, it is equally probable for increase in emotional support to indicate age-associated declines or to provide protection against the same declines. Taken together, these findings imply that size and emotional support may not predict successful aging reliably and thus explains the nonsignificant impact of these two social network characteristic on the diurnal rhythm of salivary cortisol. Although lower social support has been found to relate to reduced AUC in diurnal salivary cortisol in older adults [30], support other than emotional ones was also examined by the investigators. The significant impact on AUC might be attributed to other types of support.

 On the other hand, the health implications of attenuation of both the CAR and the diurnal decline in those having low motivation to cultivate their social relationships are less clear because this cannot be taken to indicate a defect in negative feedback due to the absence of an accentuated CAR [60]. Although absence of a significant diurnal rhythm has been shown to characterize cortisol profiles in people with PTSD [28], this should not apply to the present sample of healthy older adults. Evans et al. [33] have shown that poorer cognitive performance in older people is associated with a smaller CAR and a shallower diurnal decline, but the association between tendency to cultivate social relationships and cognitive performance in older people is still unknown. Further research is warranted to shed more light on the health correlates of this particular cortisol profile.

Despite the originality of our findings, their interpretability may be moderated by a number of limitations. First, it is not clear if cortisol levels and diurnal rhythm have been affected by health behaviors such as amount of sleep, physical activity level, and alcohol consumption because adverse health behaviors have been found to be associated with a flattened diurnal profile in a prior study [31]. Health behaviors could mediate the link between social network characteristics and cortisol levels. Second, direct health consequences of a “rejuvenated” cortisol profile to functional status in participants who were more committed to cultivate their social ties have not been demonstrated in the present study. Therefore, the most important question of whether older people having rejuvenated cortisol profiles are healthier and functionally more capable or not remains to be examined. A better understanding of the mechanisms whereby social network characteristics are translated into successful aging via cortisol will only be attained by taking into account the possible mediating effects of health behaviors and including health indices directly relevant to successful aging in future research.

## Figures and Tables

**Table 1:** Main characteristics of the study sample (*N* = 78).

	*n*	Percent
Gender		
Male	40	51.3
Female	38	48.7
Age		
59–65	9	11.5
66–72	26	33.3
73–79	30	38.5
80–86	13	16.7
Education		
No formal education	20	25.6
Primary	35	44.9
Secondary	21	26.9
Tertiary	2	2.6

**Table 2:** Means (SEM) of salivary cortisol concentrations (sqrt nmol/L) across two days in healthy older people.

	Minutes Postawakening							
	0	15	30	45	180	360	540	720
Day one	2.92 (.11)	3.09 (.12)	3.00 (.12)	3.00 (.13)	2.00 (.09)	1.84 (.08)	1.63 (.07)	1.30 (.06)
Day two	2.71 (.10)	2.97 (.12)	3.07 (.11)	2.81 (.12)	1.93 (.08)	1.83 (.07)	1.56 (.06)	1.36 (.06)

Standard errors are in parentheses. *N* = 78.

**Table 3:** Means (SEM) of salivary cortisol concentrations (sqrt nmol/L) in older people with high versus low scores in social network cultivation in the postawakening period.

	Minutes Postawakening			
	0	15	30	45
High	2.70 (.11)	3.05 (.14)	3.15 (.14)	3.05 (.14)
Low	2.93 (.14)	3.00 (.16)	2.91 (.15)	2.78(.16)

Standard errors are in parentheses. High: high scores in social network cultivation (above median score); low: low scores in social network cultivation (below median score). *N* = 78.

**Table 4:** Means (SEM) of salivary cortisol concentrations (sqrt nmol/L) in older people with high versus low scores in social network cultivation across diurnal time points.

	Hours Postawakening				
	0	3	6	9	12
High	2.70 (.11)	1.88 (.10)	1.72 (.06)	1.53 (.07)	1.24 (.06)
Low	2.93 (.14)	2.06 (.10)	1.94 (.08)	1.66 (.07)	1.42 (.08)

Standard errors are in parentheses. High: high scores in social network cultivation (above median score); low: low scores in social network cultivation (below median score). *N* = 78.
